# Extraction and Characterization of Cocoa Bean Shell Cell Wall Polysaccharides

**DOI:** 10.3390/polym15030745

**Published:** 2023-02-01

**Authors:** Amalie Younes, Salwa Karboune, Lan Liu, Eugenio Spadoni Andreani, Sarah Dahman

**Affiliations:** Department of Food Science and Agricultural Chemistry, Macdonald Campus, McGill University, Ste Anne de Bellevue, QC H9X3V9, Canada

**Keywords:** cocoa bean shells, cell wall polysaccharides, prebiotic activity

## Abstract

Cocoa bean shells (CBS), a by-product of the cocoa industry, from two cacao varieties and obtained after selected processing conditions (fermentation, drying, roasting) were characterized in terms of their chemical composition, where they were found to be a great source of carbohydrates, specifically dietary fiber, protein, ash, and polyphenols, namely quercetin, epicatechin, and catechin. Cell wall polysaccharides were isolated by alkaline extraction (0.5 M or 4 M KOH) and were found to be enriched primarily in pectic polysaccharides (80.6–86%) namely rhamnogalacturonan and arabinogalactan as well as hemi- cellulosic polysaccharides (13.9–19.4%). Overall, 0.5 M KOH polysaccharides were favored having provided a diverse profile of neutral sugars and uronic acids. When tested for the promotion of the growth of selected probiotic strains, CBS cell wall polysaccharides performed similarly or more than inulin and rhamnogalacturonan based on the prebiotic activity scores. The short-chain fatty acid profiles were characterized by high amounts of lactic acid, followed by acetic and propionic acid.

## 1. Introduction

The generation of cacao (*Theobroma cacao)* by-products is estimated at 700,000 tons annually, which negatively contributes to the environmental and economic value of the cocoa processing industry. Among these by-products, cocoa bean shells (CBS) are the most abundant and are the outermost layer of the cacao bean that encases cacao nibs. Efforts to valorize CBS have been explored in agricultural and bioremediation fields such as its use in animal feeds and fertilizers [1,2]. In food applications, CBS has been used as a functional ingredient for fiber enrichment and fat replacement in baked goods and as a stabilizer and color enhancer in pork sausages [3,4,5]. Although there has been an increasing industrial interest in effective valorization of CBS into functional food ingredients, only the pilot-scale extraction of polyphenols from CBS has been explored [6]. The extraction of cell wall polysaccharides from CBS has yet to be reported. To the authors’ knowledge, only few studies have characterized the cell wall polysaccharide profile of CBS in terms of monosaccharide profile, molecular weight distribution and linkage types [7,8,9,10]. According to [8], CBS holds a reliable source of dietary fibers as its cell-wall polysaccharide profile comprises of 45% pectic polysaccharides, 35% cellulose, as well as 20% hemicelluloses, namely gluconoarabinoxylan, xyloglucan, and galactoglucomannans. As far as the authors are aware, the extent of the variability in the cell wall polysaccharide profile of CBS, depending on the cacao variety and the processing conditions, has not been studied. 

Several approaches have been reported for the isolation of cell wall polysaccharides from cell wall, including chelating agent, acid, microwave-assisted alkaline, and ultrasound-assisted alkaline extractions [8,11,12,13]. The selection of an appropriate extracting agent or approach can help target the isolation of polysaccharides with well-defined structures. As an example, acidic extraction, using hydrochloric acid, targets the isolation of homogalacturonan [11]. Alternatively, alkaline extraction of cell wall polysaccharides, using sodium or potassium hydroxides paired with sodium borohydride, has proven to be effective for isolating and understanding the distribution of pectic and hemi-cellulosic polysaccharides within the cell wall materials of potato, cranberries, bamboo, maize, and more [11,14,15,16]. This approach enables the release of rhamnogalacturonan I through ß-elimination and oxidative peeling of homogalacturonan [11,17,18].

There is an increasing interest in the cell wall-rich by-products as their polysaccharide content can act as a beneficial source of dietary fibers and prebiotics that can modulate the gut microbiota and hence promote the intestinal health [19]. Cell wall polysaccharides of by-products derived from pearl millet fibre, tangerine peels, apple pomace, and coconut residues have been previously reported to support the effective growth of beneficial gut microorganisms [19,20,21,22]. However, no study on the fermentability of CBS cell wall polysaccharides by beneficial lactic acid bacteria has been reported so far. 

The aim of the present study was to assess the nutritional composition of CBS at various processing conditions. To the authors’ knowledge, this has not been previously investigated for cocoa beans. In addition, the present study offers novel insight pertaining to the isolation and characterization of CBS cell wall materials from two cacao varieties obtained after selected processing conditions (fermentation, drying, roasting). The cell wall polysaccharides were, thereafter, extracted by alkaline treatments, structurally characterized, and subjected to fermentation by selected lactic acid bacteria. The investigation of the compositional, structural, and functional properties of CBS cell wall polysaccharides would provide novel insight useful to the development of a highly effective approach that valorizes cocoa bean shells as a source of carbohydrate-based functional ingredients in food applications. 

## 2. Materials and Methods

### 2.1. Materials

Fresh cacao pods (CCN 51) were supplied by Smart Natural Circle (Montreal, QC, Canada). Commercial samples of CBS [Nacional/Arriba (NAC) and CCN 51] were supplied by the cocoa processing industry (Guayaquil, Ecuador). The bacterial strains, *Lactobacillus rhamnosus GG* (RO343 AR) and *Bifidobacterium longum* (ATCC 15707™) strains were supplied from Harmonium International (Mirabel, QC, Canada) and ATCC©. 1-Kestose, nytose, and 1F-fructofuranoysl-nystose were obtained from Wake Pure Chemical (Osaka, Japan). The analytical grade reagents were from Sigma- Aldrich Co., St. Louis, MO, USA, and salts were obtained from Fisher Scientific (Fair Lawn, NJ, USA). 

### 2.2. Preparation of Cocoa Bean Shells

Fresh cacao pods (CCN 51) were subjected to lab-scale fermentation and drying processes to retrieve the cacao bean shells (CBS) as described by [23]. The inner contents of cacao pods, containing the mucilage-pulp and beans, were placed in tightly sealed containers (4 L) to provide an anaerobic environment in an incubator (Excella E24 incubator, New Brunswick Scientific, NJ, USA), for 117 h, where the temperature was adjusted in 6–24 h intervals, 28 °C at 0 h, 30 °C at 6 h, 32 °C at 21 h, 35 °C at 30 h, 38 °C at 45 h, 42 °C at 54 h, and 45 °C at 69 h. The pulp-bean mass was mixed every 24 h to ensure proper removal of the pulp. After fermentation, the beans were subjected to drying in an oven for 72 h at 45 °C until a final water content of 6–7%. The beans were de-shelled manually. Commercial samples of cocoa bean shells were from two varieties [Nacional/Arriba (NAC) and CCN 51]. Both freshly prepared and commercial CBS samples were blended using a model 7011C commercial blender (Conair corporation, Stamford, CT, USA) and analyzed for their proximate compositions. 

### 2.3. Proximate Compositional Analysis of Cocoa Bean Shells

Moisture content of CBS was determined using a variation of AOAC method 927.05 by drying for 18 h at 70 °C in vacuum oven. Total fat content of blended CBS was determined using a modified AOAC method 945.16. In this last method, Soxhlet extraction of CBS (1 g with 150 mL) was carried out using petroleum ether as solvent for 6 h. Ash content was measured by AOAC method 942.05, charring 2 g of blended CBS for 30 min, followed by incineration in a muffle furnace (5 h at 525 °C). Crude protein content was determined on 0.1 g of blended CBS by Dumas method [24] with a FP 628 analyser (LECO Corporation, St Joseph, MI, USA). Carbohydrate content (% dry weight) of blended CBS will be determined as:

100−(crude fat+ash+crude protein)

Dietary fiber content was determined on 1 g of blended CBS using the total dietary fiber assay kit (Sigma-Aldrich Co., St. Louis, MI, USA) and a modified AOAC method 985.29 [25], in which α-amylase, protease and amyloglucosidase activities were used to digest the starting material. The remaining fiber was precipitated with ethanol, washed with ethanol and acetone, filtered, and, once dry, its protein and ash content were determined and subtracted from the dry weight. All analyses were performed in triplicate.

### 2.4. Isolation of Cell Wall Materials from Cocoa Bean Shells

The cell wall material (CWM) was sequentially isolated [8]. Using de-fatted CBS (4 g), the isolation proceeded as follows: (1) 80% ethanol (2 h, 50 rpm), (2) chloroform: methanol (1:1, 1.5 h, 50 rpm), (3) chloroform: methanol (1:1, 1.5 h, 50 rpm), (4) phenol: acetic acid: water (PAW, 2:1:1, overnight), (5) phenol: acetic acid: water (PAW, 2:1:1, 2 h). Each treatment was followed by centrifugation (7000× *g*, 15 min) and vacuum filtration prior to the following step. The recovered CWM was dialyzed (5–8 kDa cut-off) against distilled water and freeze-dried. The isolation of CWM was performed in triplicate. 

### 2.5. Alkaline Extraction of Cocoa Bean Shell Cell Wall Material Polysaccharides

Extraction of CBS polysaccharides was performed [11] where CWM material (2% w/v) was suspended in KOH solutions (0.5 and 4 M) containing 0.02 M NaBH_4_. Mixtures were incubated (60 °C, 24 h), thereafter centrifuged (10,000 × *g* for 15 min) and recovered via filtration (0.45 µm). Recovered polysaccharides were neutralized with HCl, dialyzed (5–8 kDa cut-off) and freeze-dried

### 2.6. Structural Characterization of Carbohydrates

#### 2.6.1. Determination of Total Neutral Sugars and Uronic Acid Contents

Uronic acid content was measured by sulphamate/m-hydroxydiphenyl assay [26]. The phenol–sulphuric acid colorimetric assay was used for the determination of neutral sugar content [27]. 

#### 2.6.2. Monosaccharide Profile

CWM and CBS polysaccharides were subjected to acid hydrolysis as previously described [11]. Briefly, 1 mL of a mixture of 37% HCl/methanol (1:4 *v/v*) was added to the sample (200 μL of liquid extract, or 5% (*w/v*) of solid in water). The mixtures were incubated at 70 °C for 24 h and dried by heating in a convection incubator (Excella E24 incubator, New Brunswick Scientific, NJ, USA). To complete hydrolysis, 3 mL of water and 1 mL of trifluoroacetic acid were added and heated at 100 °C for 1 h. After evaporating the trifluoroacetic acid, the samples were neutralized with NaOH and centrifuged (8000 rpm, 5 min). The supernatants (0.02 mL) were analyzed by high performance anion exchange chromatography (HPAEC) on a Dionex ICS 3000 system equipped with pulsed amperometric detection using a Carbopac PA-20 column (Dionex Co., Sunnyvale, CA, USA) at a temperature of 30 °C. Mobile phase was 20 mM NaOH at flow rate of 0.4 mL/min. Rhamnose, arabinose, glucose, xylose, galactose, and mannose were used at varying concentrations (2.5–50 µM) as standards.

#### 2.6.3. Molecular Weight Distribution

A high-performance size-exclusion chromatography (HPSEC) system (model 1525 binary HPLC pump, equipped with a model 2414 refractive index detector, Waters Co., Milford, MA, USA) was used to estimate the molecular weight distribution of the carbohydrates. Analysis was determined using a TSK G5000 PWXL (Tosoh Co., Yamaguchi, Japan) column, with dextrans (50–670 kDa), soybean rhamnogalacturonan (0.125–1 g/L), and galactoglucomannan (0.125–1 g/L) standards for molecular weight and concentration calibration. The temperature of the system was 30 °C, the eluent was 0.1 M NaCl, and the flow rate was set at 0.4 mL/min. 

### 2.7. Extraction and Determination of Polyphenols Content

The polyphenolic compounds were extracted using accelerated solvent extraction (Dionex ASE 350, Dionex, Sunnyvale, CA, USA) from CBS and CWM. We placed 0.5 g samples in 22 mL cells containing Ottawa sand and diatomaceous earth. Samples were subjected to three consecutive repeated extractions using 80% methanol (40 °C,1600 psi) to exhaust the material. The total polyphenolic content was determined using the folin–ciocalteu assay as previously described [28]. A gallic acid calibration curve (0.001 to 0.1 mM) was constructed. The total polyphenolic content was expressed as gram of gallic acid equivalent per gram of sample. All measurements were performed in triplicate.

### 2.8. Determination of Phenolic Compounds via LC-MS

CBS and CWM were analyzed by LC-MS using an Agilent 1290 Infinity II LC system coupled to the 6560-ion mobility Q-TOF-MS (Agilent Technologies, Santa Clara, CA, USA). The LC separation was conducted on a Poreshell120 EC-C18 analytical column (Agilent Technologies; 2.7 μm × 3 mm × 100 mm) connected with a Poreshell120 EC-C18 guard column (Agilent Technologies; 2.7 μm × 3 mm × 5 mm). The mobile phase A was HPLC water with 0.1% formic acid and the mobile phase B was acetonitrile with 0.1% formic acid. HPLC parameters were as follows: injection volume was 1 µL, the flow rate was 0.3 mL/min, and the column temperature was set to 30 °C. The mobile phase profile used for the run in negative ion mode was 2% B (0–1.0 min), 2%–20% B (1.0–4.0 min), 20%–100% B (4.0–8.0 min), 100% B (4.0–8.0 min), hold at 100% B (8.0–13.0 min), decrease to 2% B (13.0.0–13.5 min), and hold 2% B (13.5–14 min). The mass spectrometer was equipped with a dual AJS ESI ion source operating in negative ionization mode. MS conditions were as follows: for ESI-, the drying gas temperature was 200 °C, drying gas flow rate was 12 L/min, sheath gas temperature was 250 °C, sheath gas flow rate was 12 L/min, the pressure on the nebulizer was 35 psi, the capillary voltage was 4000 V, the fragmentor voltage was 240 V, and the nozzle voltage was 1000 V. Full scan MS data were recorded between mass-to-charge ratios (*m/z*) 100 and 1100 at a scan rate of 2 spectra/s, and were collected at both centroid and profile mode. Reference ions (*m/z* at 112.9856 and 1033.9881 for ESI-) were used for automatic mass recalibration of each acquired spectrum. Data treatment was conducted using Quantitative Analysis B.07.01 from Agilent MassHunter Workstation Software.

### 2.9. Prebiotic Activity Assay

The fermentability of CBS polysaccharides (0.5 M KOH extracts, roasted CCN and NAC) by *B. longum* (ATCC 15707™) and *L. rhamnosus GG* (Harmonium AR) was investigated and compared to that of inulin and rhamnogalacturonan, using a modification of the method reported by [29]. The selected strains, *B. longum* and *L. rhamonosus GG,* were maintained in an anaerobic chamber at 37 °C. Pre-reduced reinforced clostridial broth was used for the reanimation of *B. longum* from a freeze-dried stock, and MRS broth was used for *L. rhamnosus GG*. An aliquot (0.5 mL) of the primary cultures was then added into tubes containing 5 mL of pre-reduced, carbohydrate-free MRS broth, supplemented with 0.4% glucose. Bacterial growth was measured using colony counting on Tryptic soy agar supplemented with 5% sheep blood for *B. longum* and pre-reduced MRS agar for *L. rhamnosus GG.* Tubes containing 5 mL of pre-reduced carbohydrate-free MRS broth supplemented with 0.2% glucose, inulin, rhamnogalacturonan, and cell wall polysaccharides (CCN51 and NAC varieties) were inoculated with 0.1 mL of secondary culture. Colonies were counted on Tryptic soy agar supplemented with 5% sheep blood for *B. longum and* pre-reduced MRS agar for *L. rhamnosus GG* after 6, 24, 48, 72, and 96 h incubation intervals. Microbial counts were measured as colony forming units (CFU)/mL, and the resulting averaged data were transformed to log CFU/mL. A prebiotic activity score (PA) was calculated for each combination of bacterium and polysaccharide as follows:

$$\mathrm{PA}=\frac{\left(\mathrm{Log}\frac{\mathrm{CFU}}{\mathrm{ml}}\mathrm{on}\;\mathrm{polysaccharide}\;\mathrm{at}\;x\;\mathrm{hr}-\mathrm{Log}\frac{CFU}{ml}on\;polysaccharide\;at\;0\;hr\right)}{Log\frac{CFU}{ml}on\;glucose\;at\;x\;hr-Log\frac{CFU}{ml}on\;glucose\;at\;0\;hr}$$

### 2.10. Determination of Short Chain Fatty Acid Catabolites 

An aliquot of cultures (0.5 mL) was taken at 24 and 48 h. Bacteria were precipitated by centrifugation at 1400× *g* for 5 min at 37 °C in a Minispin Plus centrifuge (Eppendorf AG, Hamburg, Germany). The supernatants were filtered using 0.22 µm polytetrafluorethylene syringe filters (Thermo Fischer Scientific) and stored at −80 °C for a maximum of 2 weeks. Freshly thawed samples (0.02 mL) were analyzed using the HPLC model 1525 system (Waters) equipped with a refractive index detector and a Zorbax SB-C18 column (4.6 × 250 mm) (Agilent Technologies). Elution was done with 0.005 N sulfuric acid at a constant flow rate of 0.6 mL/min. Standard curves were constructed using lactic, acetic, propionic, and butyric acids as standards. 

### 2.11. Statistical Analysis

Statistical analyses were performed using XLSTAT software (Addinsoft, New York, NY, USA) in Microsoft Excel (Microsoft, Redmond, WA, USA). One-way analysis of variance (ANOVA) and the Tukey’s honest significant difference (HSD) test were performed to detect significant differences (*p* < 0.05). 

## 3. Results and Discussion

### 3.1. Characterizaton of Cocoa Bean Shells and Its Cell Wall Material

The chemical composition of fermented (CCN), dried (CCN), and roasted (CCN and NAC) cocoa bean shells was determined based on AOAC standard protocols. Overall, the composition of CBSs comprised of 12.7–19.7% protein, 0.3–7.6% fat, 6.1–7.1% ash, 65.5–80.7% carbohydrates, and 50.1–61.4% fibers (Table 1). These findings are within the ranges reported in the literature [30,31,32,33,34], which found CBS to comprise of 6.2–18.6% protein, 2.02–6.46% fat, 6–11.67% ash, 17.8–55.85% carbohydrates, and 13.86–60.6% fibers. However, it should be noted that the broad range in the composition is due to variations in their origin, variety, processing condition, and assessment method. 

The protein content has shown a noticeable increase (*p* < 0.05) from its fermented (13%, CCN) to dried (18.9%, CCN) states, followed by a decrease in its roasted state (12.8%, CCN). According to [31], the observed increase in the protein content from fermented to dried conditions is attributed to the increased microbial mass which produces nitrogen during growth”. The decrease in protein content, following roasting, may be a result of the elevated temperatures involved in the roasting process (120–150 °C). This mediates the Maillard reaction, which involves the interaction between the carbonyl group of a reducing sugar and a free amino acid from protein, thereby decreasing the protein content [31,35]. Interestingly, only 1% of the protein content in cocoa bean shells exists in its free form [34]. The remainder is strongly bound to oxidized polyphenols, further converting into polyphenol–quinones. Similarly, the fat content increases (*p* < 0.05) with the processing of cocoa beans from fermentation to drying and roasting. The variation in the fat content can be explained by the subjection of elevated temperatures involved in the drying and roasting processes (70–150 °C), further inducing the migration of cocoa butter, from its cotyledon layer to the cocoa bean shells [36,37]. The fatty acid composition in CBS has been reported to predominantly contain palmitic and oleic and linoleic acids [37].The ash content (*p* < 0.05) is relatively stable amongst the four samples, fermented, dried, and roasted CBS. Overall, cocoa bean shells have shown to contain higher ash contents than other fruit by-products, namely passion fruit seeds (1.34%), and apple pomace (0.5%) [32,38,39]. 

On average, the fiber content in cocoa bean shells accounts for around 50% of the entire material. Our findings are consistent with the literature [6,40] in which all four CBS samples contain 50.1–64.1% fiber. Using the sequential isolation approach, involving the use of chloroform and methanol, ethanol, followed by phenol, acetic acid, and water (2:1:1), to retrieve the cell wall material, we observed an overall cell wall material yield between 40.6 and 48.1%; higher than previously reported [8]. The overall ratio between neutral sugar and uronic acid between the fermented and dried shells (0.82–0.97) compared to the roasted shells (1.4–1.8), indicate a higher proportion of neutral sugars upon subjection to roasting. One study observed a reduction in the cell wall polysaccharide composition of apple chips, specifically fucose, rhamnose, arabinose, and xylose, with increased drying temperatures (60–90 °C); further supporting that temperature conditions significantly influence the neutral sugar and uronic acid profile of foodstuff [41]. 

Based on the findings, as determined by anionic exchange chromatography (Table 1), all four sets of CBS CWM, regardless of their processing conditions, were predominantly rich in pectic polysaccharides (80.6–86%), comprised of uronic acids (35.1–50.5%), galactose (22.7–29.2%), arabinose (13.6–17.2%), and rhamnose (5.1–6.9%) residues. The remainder consists of hemi-cellulosic polysaccharides (13.9–19.4%), comprised of glucose (3.3–7.1%), fucose (2.4–3.6%), xylose, and mannose (0.9–1.9%) residues. These are consistent with the other works [6,8] which suggested that CBS contain a greater proportion of pectic and non-cellulosic cell wall polysaccharides than cellulosic polysaccharides. Upon extracting the cell wall material, the protein content (12.9–16.6%) remained unchanged upon extraction, likely indicating the complexes between dietary fiber and protein from the Maillard reaction, especially in the roasted samples [8]. 

### 3.2. Polyphenolic Profile of Cocoa Bean Shells and Its Cell Wall Material

Polyphenolic compounds are naturally present within the cotyledons of the cocoa seeds; however, compounds can migrate towards the shell through diffusion, further enriching the by-product [34,42,43]. The CBS total polyphenol content comprised between 0.4 and 0.6% (Table 1) similar to the content found in cocoa beans [44,45]. In addition, after isolating its CWM, the total polyphenol content was obtained around 0.05–0.1%, directly influenced by the use of solvents in the sequential isolation process used to retrieve the CWM. In general, the polyphenol content is expected to increase with further processing. Our findings showed no significant differences in the polyphenol content of CBS at different processing conditions of fermentation, drying and roasting. However, overall, the roasted CBS samples (NAC and CCN) hold a greater total polyphenol content as compared to their fermented and dried states. Alternatively, dried CBS polyphenols may be more tightly bound to cell wall materials than fermented and roasted CBS. Subjection of heat from the drying and roasting process can mediate not only the Maillard reaction, but also promote condensation and polymerization. Given our findings showed a great amount of crude fiber (non-digestible carbohydrates and lignin), one may suggest that lignin is pyrolyzed slowly with the onset of roasting conditions allowing for the release of polyphenols entrapped within its network, otherwise known as the polyphenol complex [31,46,47].

Based on our findings, as determined by LC-MS (*p* < 0.05) in Table 1, the most abundant class of polyphenols found in CBS and its CWM are the flavan-3-ols, which include epicatechin (122.8–0.9 ng/kg material or extract) and catechin (24.3–0.3 ng/kg material or extract; this is consistent with the literature [48,49]. Other polyphenols were detected in both CBS and the corresponding CWM, which include flavonoids, such as quercetin, as well as phenolic acids, such as gallic, protocatechuic, p-coumaric, ferulic, and caffeic acids. Nevertheless, cocoa and its by-products are known for its richness in methylxanthines, such as theobromine and caffeine [34]. Our findings detected considerable amounts of caffeine among all samples of CBS and its CWM; with the highest amounts found among the fermented and dried types (139.4–261.6 ng/kg material). Interesting, the caffeine content was significantly lower after the roasting process. According to [46], caffeine and other polyphenols were found in greater amounts in their raw and lightly roasted forms (160 °C); further indicating the effect of processing conditions on the polyphenolic profile. 

### 3.3. Extraction of Cell Wall Polysaccharides and Their Characterization

To extract and characterize the CBS cell wall polysaccharides, alkaline extraction was performed using two concentrations of KOH, 0.5 M and 4 M. The polysaccharide yield (Table 2) ranged between 23.7 and 35.4% (*w/w*) using 0.5 M KOH depending on the starting materials (fermented, dried, roasted) and variety. Interestingly, as the alkaline solution increased, the roasted samples (CCN and NAC) saw an increase in their polysaccharide yields (32.9–53.6%, *w/w*). Such findings are consistent with the findings assessed on pectic polysaccharides derived from potato peel and cranberry pomace [11,14]. In addition, the results show the proportion of neutral sugars decreased as the alkaline solution was increased, whereas the molar proportion of uronic acid remained relatively unchanged. As a result, the uronic/neutral sugar ratio increased, possibly indicating the onset of debranching or defragmentation of neutral sugar regions.

The monosaccharide composition (Table 2) reveals the main sugars in the extracted CBS cell wall polysaccharides were uronic acids (42.9–58.2%), arabinose (7.8–16.4%), and galactose (8.1–22.4%) for the 0.5 M alkaline treatment, signifying the presence of arabinan, galactan, and rhamnogalacturonan. As for the 4 M alkaline treatment, the main sugars were uronic acids (56.4–66.7%), galactose (9.9–15.9%), xylose, and mannose (2.6–15.9%), indicating a higher proportion of hemicellulose as compared to the latter. We can estimate the proportion of pectic polysaccharides by determining the pectic neutral sugars content, calculated as the ratio of rhamnose, arabinose, and galactose to total neutral sugars. The results suggest the polysaccharide profile of CBS alkaline extracts are predominantly pectic polysaccharides (56.4–88.8%) with the remainder being hemi-cellulosic polysaccharides, as signified by the presence of xylose, mannose, and glucose. Using a 4 M KOH extraction treatment, [8] reported the presence of a mixture of hemicelluloses and pectic polysaccharides based on the levels of uronic acid (26.2%) xylose (23.5%), glucose (21.8%), and mannose (9.9%). In addition, the study reported rhamnogalacturonan as the main pectic polysaccharide, as well as three hemi-cellulosic polysaccharides, namely galactoglucomannan, xylogalacturonan, and glucoarabinoxylan [8]. These findings are supported by the fact that pectic neutral sugars are of a lower proportion compared to 0.5 M alkaline extracts, comprising 55.6–76.1% (Table 2). Considering all samples (using both treatments) showed high proportions of pectic polysaccharides, the ratio of arabinose and galactose to rhamnose provides an estimate of the level of branching of rhamnogalacturonan I. As seen in Table 2, the 4 M extracts showed the highest ratios, indicating the presence of more abundant arabinan and galactan side chains. Furthermore, by increasing the alkaline solution concentration, the degree of branching increased by two to five-fold, depending on the processing condition. Based on the rhamnose molar proportions with the 0.5 M alkaline treatment, it can be assumed that rhamnogalacturonan I is more abundant in fermented and roasted CCN 51 samples.

As the alkaline solution increased, the molar proportion had decreased to 2.3–3.7%. All in all, the NAC variety attained a higher polysaccharide yield (35.4–53.6%) among the two alkaline treatments compared to the CCN variety (19.5–32.9%). In addition, the polysaccharide profiles of both varieties showed similarities, with the exception that the CCN variety expressed a richer profile of galactose (10.7–15.9% µM) and xylose and mannose (4.2–10.6% µM), whereas the NAC variety expressed a richer content of arabinose (13.4% µM). 

**Figure 1:**
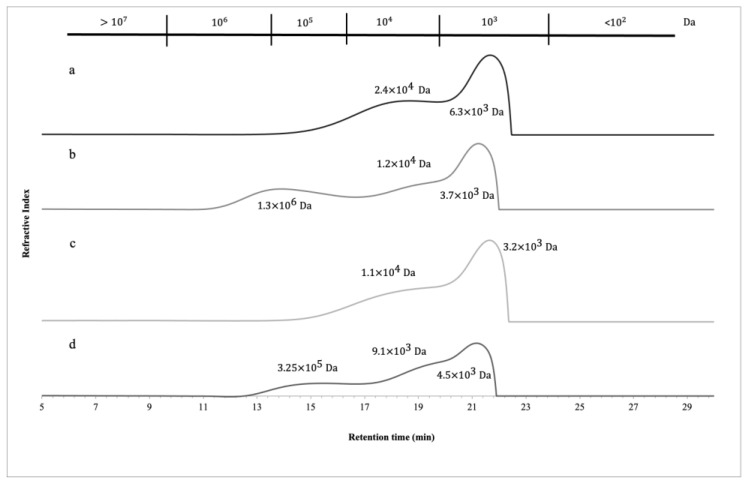
Molecular weight distribution of CWP alkaline extracts. Letters are denoted by the different varieties (NAC, CCN) subjected to different alkaline solution treatments (0.5 M, 4 M KOH) A: NAC 4 M, B: NAC 0.5 M, C: CCN 4 M, D: CCN 0.5 M, respectively.

Roasted samples (NAC and CCN varieties) were analyzed for their molecular weight (MW) distribution. The results showed a significant impact of increasing the alkaline solution from 0.5 to 4 M KOH. In the 0.5 M extracts, three main MW populations were found at 3.7, 12, and 130, kDa (NAC variety) and 4.5, 9.1, and 32.5 kDa (CCN variety) (Figure 1). Increasing the alkaline solution resulted in a shift from high to low molecular weight fractions, where the 4 M extract main populations were found at 24 and 6.3 kDa (NAC variety) and at 11 and 3.2 kDa (CCN variety) (Figure 1). This is a result of increasing the alkaline solution, which allowed for a higher degree of debranching or defragmentation of the pectic polysaccharides present in CBS. As per the author’s knowledge, no studies have been reported on the MW populations of CBS. However, [7] previously reported that a high MW population (2000 kDa) was detected in cell wall polysaccharides extracted from unfermented and fermented cocoa beans. The MW populations of cranberry pomace (1.5–1500 kDa), potato peel protein (1–600 kDa), and olives (260–400 kDa) have also been reported, showing a profile mixture of both low and high MW fractions [11,14,50]. The presence of low MW fractions may be due to the disassociation of non-covalently bound oligosaccharides during the dialysis purification steps [14]. 

### 3.4. Prebiotic Activity

**Figure 2:**
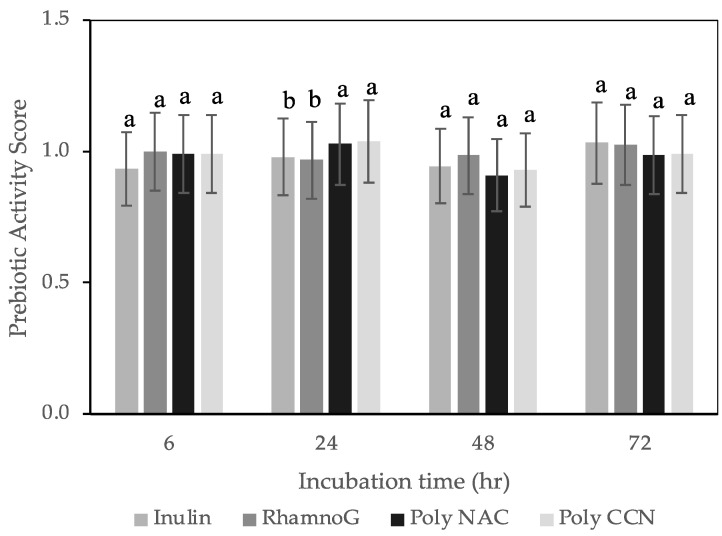
Prebiotic activity scores of *Lactobacillus rhamnosus GG* (R0343 AR) on polysaccharide extracts, inulin and rhamnogalacturonan, measured at 6,24, 48, 72, and 96 h of incubation. Values represented as an average ± standard deviation. Poly NAC: polysaccharide NAC variety, poly CCN: polysaccharide CCN variety, RhamnoG: Rhamnogalacturonan, respectively. For each incubation time, bars with different letters represent scores significantly different at *p* < 0.05.

**Figure 3:**
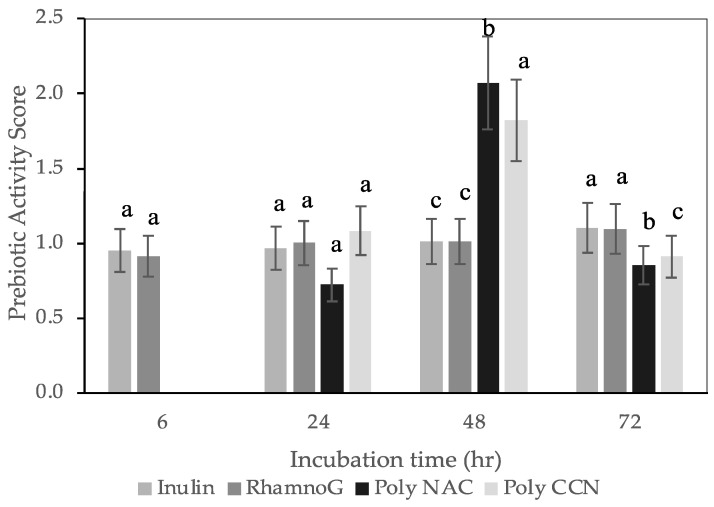
Prebiotic activity scores of *Bifidobacterium longum* (ATCC^®^ 15707) on polysaccharide extracts, inulin and rhamnogalacturonan, measured at 6, 24, 48, 72 and 96 h of incubation. Values represented as an average ± standard deviation. Poly NAC: polysaccharide NAC variety, Poly CCN: polysaccharide CCN variety, RhamnoG: Rhamnogalacturonan, respectively. For each incubation time, bars with different letters represent scores significantly different at *p* < 0.05.

The prebiotic activity scores of CBS polysaccharides, rhamnogalacturonan, and inulin, a known prebiotic polysaccharide [51], were compared using them as carbon sources for the anaerobic growth of two probiotic bacteria strains, *L. rhamnosus GG* and *B. longum.* Both strains were handled and grown in anaerobic conditions. While *L. rhamnosus GG* is oxygen-tolerant, the anaerobic conditions were selected to simulate the intestinal environment more accurately. In general, *Lactobacillus* species show a significant heterogeneity in their abilities to ferment carbohydrates [19,52,53,54]. Figure 2 shows that CBS polysaccharides (NAC and CCN varieties) showed a prebiotic activity score similar to that of inulin and rhamnogalacturonan for *L. rhamnosus GG* (Figure 2). At 24 h of fermentation, the prebiotic activity scores of CBS polysaccharides were significantly higher (*p ≤* 0.05) than that of inulin and rhamnogalacturonan. For *B. longum* (Figure 3), CBS polysaccharides showed statistically significant (*p ≤* 0.05) prebiotic activity scores at 48 h of fermentation than that of inulin and rhamnogalacturonan; however, the prebiotic activity scores decreased at the 72 h fermentation period. Therefore, it appears that CBS polysaccharides, primarily rich in pectin, galactan, and arabinan, effectively stimulate the growth of both strains. Dietary fibers, rich in pectin, undergo bacterial fermentation in the ileum and colon, leading to acidification of the colonic contents as well as the production of short-chain fatty acids [55,56,57]. Studies reported effective growth of *L. rhamnosus GG* with pectic polysaccharides and galacto-oligosaccharides derived from pearl millet fibre, and *F. kuhistanic* leaves [20,21] and *B. longum* with pectic polysaccharides derived from cranberry pomace, pumpkin peel, and citrus pectin [19,58,59].

Interestingly, gene expression analysis of *B. longum* indicate the presence of over 40 glycosyl hydrolases, which include two xylanases, nine arabinosidases, two α-galactosidases, and more, whose predicted substrates cover a wide range of di, tri-, and higher order oligo-saccharides, indicating the strain’s high affinity for oligosaccharides with a degree of polymerization less than 8 [60].

Regarding, *L. rhamnosus GG*, this strain has shown the highest affinity towards fucosylated oligo- and polysaccharides as it has been found to express the fucose permease, fucose isomerase, fucolose kinase, fucose mutarotose, and fucolose-1-phosphate aldolase, promoting the fucose catabolic pathway to produce 1,2-propanediol or lactate [61]. According to one study assessing the probiotic activities of *B. longum* and *L. rhamnosus GG* using qPCR showed that both strains indirectly utilize pectic polysaccharides as a source of prebiotics. The polysaccharides undergo metabolization prior by other gut microbiota, namely *E. coli* H, resulting in metabolic end products that are then metabolized by *B. longum* and *L. rhamnosus GG* [62]. In addition, studies suggest the degree of methylation of PS may affect the fermentation rate. Based on FTIR and NMR analysis, CBS pectic fractions have been characterized as highly acetylated low methyoxyl homogalacturonan and type I rhamnogalacturonan with galactan or arabinogalactan side chains [63]. In addition, [64] reports that *Lactobacillus* strains showed higher fermentation rates with polysaccharides with a low degree of methylation. This may explain the faster fermentation rates observed with *L. rhamnosus GG* with CBS polysaccharides. Studies also found conflicting correlations between the degree of esterification and bacterial taxa in stimulating their growth [64,65,66]. Further investigation on the structural linkage, degree of methylation and degree of esterification is needed to assess whether they could result in the differential promotion of the growth of selected bacteria. 

SCFA are produced upon the fermentation of dietary fiber by gut microbiota [19,67]. Prebiotic production of SCFA can be used to complement *L. rhamnosus GG* and *B. longum* fermentation of CBS polysaccharides. Table 3 and Table 4 show the concentration of four SCFA after 24 and 48 hr of fermentation of *L. rhamnosus GG* and *B. longum* with glucose, CBS polysaccharides (NAC and CCN varieties) and positive controls, inulin and rhamnogalacturonan. In the culture of *L. rhamnosus GG* (Table 3)*,* the main increase was seen in the lactic acid followed by acetic acid. Although unabundant, the significance (*p* < 0.05) was observed with the production of propionic acid for CBS polysaccharides from both varieties (NAC and CCN) at 48 hr of fermentation. *L. rhamnosus GG* is known for its ability to produce lactic and propionic acid during fermentation, which can promote anti-inflammatory and anti-microbial properties in our gastrointestinal tract [68,69]. The production of butyric acid was prevalent in glucose, inulin, and rhamnogalacturonan, indicating that CBS polysaccharides do not favor the production of butyrate in the *L. rhamnosus* fermentation pathway. However, the presence of butyric acid can be attributed to the metabolization of lactate, further explaining the decrease in the lactate concentration with increased incubation time [70,71]. 

Similar to the SCFA profile of *L. rhamnosus GG,* the culture of *B. longum* was characterized by the greatest increase in lactic acid, followed by acetic acid predominantly after 48 h of fermentation with the except of CCN polysaccharides. The acetic acid content of the medium remained relatively unchanged for the polysaccharides. The lactic acid concentrations produced by polysaccharides were higher at 24 h of fermentation than that of glucose, inulin, and rhamnogalacturonan. At 48 h of fermentation, the lactic acid concentrations decreased and were surpassed by their positive controls. However, propionic, and butyric acid concentrations either decreased or were not detected after 24 h of *B. longum* fermentation. Overall, the total SCFA profile of polysaccharides were higher at 24 h than 48 h, indicating possible SCFA degradation. Studies suggest that *Bifidobacterium* spp., specifically. *B. longum,* are producers of lactate, acetate, and propionate in the human intestinal tract [68,72]. However, only the production of lactic and acetic acids was observed with CBS polysaccharides. In addition, when carbohydrates are in excess, *Bifidobacterium* utilize the fermentation pathway, to produce two molecules of acetate with 1 molecule of lactate [68,73,74]. However, our findings suggest a higher proportion of lactic than acetic acid.

Overall, comparing the total SCFA released at 48 hrs revealed that CBS polysaccharides positively contributed towards effective growth of both strains, producing high proportions of lactic and acetic acid, compared to the growth initiated by glucose, inulin, and rhamnogalacturonan. New research suggests that multi-strain probiotics can contribute to more enhanced health benefits compared to single-strain probiotics [75,76]. Further investigation can assess the effect of multi-culturing, using *L. rhamnosus GG* and *B. longum,* to determine whether a synergistic approach can promote higher prebiotic activity scores and SCFA production at faster fermentation rates. 

## 4. Conclusions

The chemical composition of CBS (fermented, dried, and roasted) is predominantly carbohydrates (65.5–80.7%), of which around 50% is dietary fiber. CBS was also found to contain a great source of protein (12.7–19.7%) and ash (6.1–7.1%). As expected, the fat content (0.3–7.6%) was less than the amounts reported for cocoa beans; however, fat migration from the bean to the outer shell is induced upon subjection to high temperatures involved in the drying and roasting processes. Upon isolation of the CBS CWM, the polysaccharide profile was found to contain both pectic (80.6–86%) and hemi-cellulosic (13.9–19.4%) polysaccharides. Furthermore, the protein content of CWM remained unchanged relative to their raw states, indicating possible interactions with dietary fiber. The cell wall polysaccharides were isolated from CWM using two concentrations of KOH: 0.5 M and 4 M. The less concentrated extract, although it hada lower recovery yield, was favored as it provided a better neutral sugar and uronic acid profile. By increasing the alkaline concentration, defragmentation and debranching occurred. Overall, the nature of the CBS cell wall composition was found to be predominantly rich in pectic polysaccharides, such as rhamnogalacturonan. When tested for the promotion of the growth of selected probiotic strains, CBS cell wall polysaccharides performed similarly or more than inulin and rhamnogalacturonan based on the prebiotic activity scores. The short chain fatty acid profiles were characterized by high amounts of lactic acid, followed by acetic and propionic acid. Further investigation of the use of these polysaccharides using other single-strain and possibly multi-strain probiotics is needed to assess the effect of CBS polysaccharides on the selectivity of colonic fermentation.

## Figures and Tables

**Table 1:** Proximate composition of cocoa bean shells (CBS) and saccharide profile of their corresponding cell wall materials (CWM).

Proximate Analyses of CBS				
Components	Content (%, w/dry w)			
	Fermented CCN51	Dried CCN 51	Roasted CCN 51	Roasted NAC
Protein	13 ± 2 ^b^	18.9 ± 1.0 ^a^	12.8 ± 1.1 ^b^	19.8 ± 1.9 ^a^
Fat	0.3 ± 0.04 ^c^	2.3 ± 0.4 ^b^	2.1 ± 0.4 ^b^	7.6 ± 0.4 ^a^
Ash	6.1 ± 0.07 ^b^	7.1 ± 0.1 ^a^	6.5 ± 0.5 ^ab^	7.1 ± 0.3 ^a^
CHO	80.6 ± 2.1 ^a^	71.7 ± 1.6 ^b^	78.7 ± 1.1 ^a^	65.5 ± 1.6 ^c^
Fiber	61.4 ± 8.3 ^a^	50.2 ± 7.3 ^a^	50.1 ± 0.3 ^a^	54.3 ± 2.8 ^a^
Polyphenol content *	0.5 ± 0.1 ^a^	0.6 ± 0.1 ^a^	0.6 ± 0.1 ^a^	0.4 ± 0.02 ^a^
Composition of CWM of CBS				
CWM yield	40.6 ± 12.6	41.6 ± 2.6	49.4 ± 0.04	48.1 ± 3.2
Polyphenol content *	0.05 ± 0.002 ^c^	0.1 ± 0.01 ^a^	0.1 ± 0.01 ^b^	0.1 ± 0.01 ^b^
Protein content *	16.6 ± 0.8 ^a^	17.6 ± 1.6 ^a^	17.3 ± 1.4 ^a^	12.9 ± 1.1 ^a^
Neutral sugar content *	31.4 ± 5.3 ^c^	29.9 ± 7.6 ^bc^	41.9 ± 5.8 ^a^	40.9 ± 8.0 ^ab^
Uronic acid content *	32.5 ± 7.6 ^a^	36.5 ± 14.7 ^a^	22.8 ± 2 ^a^	29.8 ± 2.7 ^a^
Monosaccharide profile (%, g/100 g rel. proportion)				
Fucose	2.6 ± 0.03 ^a^	3.6 ± 2.4 ^a^	2.4 ± 0.05 ^a^	2.8 ± 1.6 ^a^
Rhamnose	6.2 ± 0.9 ^ab^	5.1 ± 1.1 ^b^	6.9 ± 0.4 ^ab^	6.4 ± 0.1 ^a^
Arabinose	13.8 ± 2.1 ^a^	13.6 ± 1.26 ^a^	17.2 ± 1.1 ^a^	14.2 ± 0.9 ^a^
Galactose	22.7 ± 2.5 ^a^	23.7 ± 3.9 ^a^	29.2 ± 1.4 ^a^	27.2 ± 0.7 ^a^
Glucose	3.3 ± 0.1 ^b^	5.2 ± 0.7 ^b^	7.1 ± 0.8 ^a^	5.9 ± 0.3 ^a^
Xylose + Mannose	0.9 ± 0.3 ^a^	1.3 ± 0.12 ^a^	1.9 ± 0.15 ^a^	1.5 ± 0.2 ^a^
Uronic acids	50.5 ± 5.9 ^a^	47.5 ± 1.1 ^a^	35.1 ± 2.0 ^a^	42.1 ± 2.2 ^a^
Hemi-cellulosic PS *	13.9 ± 0.8 ^a^	19.4 ± 6.1 ^a^	17.8 ± 1.9 ^a^	17.5 ± 2.9 ^a^
Pectic PS *	86.0 ± 0.8 ^a^	80.6 ± 6.1 ^a^	82.2 ± 1.9 ^a^	82.5 ± 2.9 ^a^
Polyphenol Profile of CBS and CWM (ng/Kg CBS/CWM CBS)				
Gallic acid	0.2 ± 0.01 ^ab^/0.3 ± 0.03 ^a^	0.2 ± 0.06 ^a^/0.2 ± 0.02 ^ab^	0.1 ± 0.02 ^bc^/n.d. **^c^	n.d. **^c^/n.d. **^c^
Protocatechuic acid	7.2 ± 1.9 ^c^/9.3 ± 0.03 ^c^	23.2 ± 3.9 ^b^/34.6 ± 5.2 ^a^	7.0 ± 0.5 ^cd^/5.3 ± 0.3 ^cd^	1.3 ± 0.1 ^d^/7.5 ± 1.1 ^c^
Catechin	4.1 ± 0.4 ^cd^/6.4 ± 0.1 ^cd^	24.3 ± 6.7 ^a^/20.2 ± 0.9 ^ab^	0.3 ± 0.06 ^d^/0.4 ± 0.04 ^d^	13.0 ± 1.3 ^bc^/2.1 ± 0.4 ^d^
Epicatechin	37.4 ± 0.1 ^bc^/65.8 ± 1.7 ^ab^	122.8 ± 7.5 ^a^/124.4 ± 26.9 ^a^	6.1 ± 0.5 ^bc^/10.1 ± 0.7 ^bc^	0.9 ± 0.1 ^c^/19.8 ± 2.6 ^bc^
Caffeic acid	0.1 ± 0.03 ^b^/0.4 ± 0.1 ^b^	0.3 ± 0.1 ^b^/0.4 ± 0.1 ^b^	0.1 ± 0.003 ^b^/9.6 ± 0.9 ^b^	1.1 ± 0.1 ^a^/0.3 ± 0.1 ^b^
p-Coumaric acid	0.05 ± 0.01 ^b^/0.05 ± 0.0005 ^b^	0.1 ± 0.01 ^ab^/0.1 ± 0.02 ^ab^	n.d. **^b^/n.d. **^b^	0.1 ± 0.01 ^ab^/0.2 ± 0.04 ^a^
Ferulic acid	0.2 ± 0.01 ^a^/0.2 ± 0.04 ^a^	0.1 ± 0.04 ^a^/0.05 ± 0.0001 ^a^	0.4 ^a^/0.3 ± 0.2 ^a^	0.2 ± 0.1 ^a^/1.2 ± 0.1 ^a^
Quercetin	2.1 ± 0.1 ^bc^/4.6 ± 0.5 ^a^	3.1 ± 0.3 ^ab^/6.0 ± 1.1 ^a^	0.4 ± 0.04 ^d^/0.5 ± 0.003 ^d^	0.8 ± 0.1 ^cd^/n.d. ** ^d^
Caffeine	261.6 ± 1.2 ^a^/257.6 ± 7.9 ^a^	139.4 ± 3.9 ^a^/169.5 ± 13.1 ^a^	4.5 ± 0.2 ^a^/19.3 ± 0.4 ^a^	17.7 ± 0.1 ^a^/22.3 ± 5.0 ^a^

Values reported as average ± standard deviation. * Units expressed as % weight per weight CWM. ** not detected. ^a^ Within the same row, means with different letters are significantly different at *p* < 0.05.

**Table 2:** Alkaline extraction of polysaccharides from the cell wall material (CWM) of cocoa bean shells (CBS).

	Fermented CCN51	Dried CCN 51	Roasted CCN 51	Roasted NAC
**0.5 M KOH**				
PS yield (%, *w/w*)	23.7 ± 0.01	24.4 ± 0.05	29.1 ± 0.4	35.4 ± 4.3
Neutral sugar content *	27.1 ± 1.9 ^a^	25.5 ± 3.2 ^a^	20.6 ± 0.7 ^b^	13.5 ± 2.3 ^b^
Uronic acid content *	54.5 ± 9.3 ^a^	55.1 ± 0.9 ^a^	58.3 ± 5.2 ^a^	49.8 ± 3.02 ^a^
Uronic/Neutral Sugar ratio	2.01	2.16	2.83	3.69
Monosaccharide Composition (%, g/100 g–relative proportion)				
Fucose	0.9 ± 0.2 ^b^	0.7 ± 0.07 ^b^	1.8 ± 0.02 ^a^	1.8 ± 0.1 ^a^
Rhamnose	11.9 ± 0.6 ^a^	9.9 ± 0.8 ^a^	12.4 ± 0.3 ^a^	6.8 ± 1.1 ^b^
Arabinose	16.4 ± 0.9 ^a^	13.9 ± 0.8 ^a^	8.6 ± 0.01 ^b^	7.8 ± 0.6 ^b^
Galactose	22.4 ± 0.9 ^a^	18.5 ± 0.4 ^b^	8.1 ± 0.2 ^c^	17.5 ± 1.3 ^b^
Glucose	1.1 ± 0.1 ^b^	3.7 ± 1.1 ^a^	5.2 ± 0.5 ^a^	2.9 ± 0.1 ^ab^
Xylose+ Mannose	4.4 ± 0.9 ^b^	8.5 ± 1.9 ^ab^	12.6 ± 2.0 ^c^	5.0 ± 0.4 ^b^
Uronic acids	42.9 ± 2.5 ^b^	44.9 ± 2.6 ^b^	51.4 ± 3.1 ^ab^	58.2 ± 2.8 ^b^
Pectic neutral sugars *	88.8 ± 1.7 ^a^	76.8 ± 4.2 ^b^	59.9 ± 2.8 ^c^	76.8 ± 1.8 ^b^
Branching	3.3	3.3	1.3	3.7
**4 M KOH**				
PS yield (%, w/w)	19.5 ± 0.05	21.1 ± 0.07	32.9 ± 0.08	53.6 ± 8.1
Neutral sugar content *	13.9 ± 2.1 ^a^	12.3 ± 0.7 ^a^	11.4 ± 1.4 ^a^	10.7 ± 0.2 ^a^
Uronic acid content *	47.9 ± 5.1 ^a^	60.3 ± 9.3 ^a^	60.8 ± 3.6 ^a^	51.6 ± 1.4 ^a^
Uronic/Neutral Sugar ratio	3.44	4.89	5.31	4.84
Monosaccharide Composition (%, g/100 g–relative proportion)				
Fucose	1.2 ± 0.2 ^b^	1.8 ± 0.1 ^b^	4.3 ± 0.1 ^a^	3.4 ± 0.4 ^a^
Rhamnose	3.5 ± 0.1 ^ab^	2.3 ± 0.4 ^b^	3.7 ± 0.3 ^a^	3.7 ± 0.5 ^a^
Arabinose	5.9 ± 0.7 ^c^	4.2 ± 0.3 ^c^	10.6 ± 0.6 ^b^	13.4 ± 0.6 ^a^
Galactose	14.9 ± 1.8 ^a^	15.9 ± 0.4 ^a^	10.7 ± 0.4 ^b^	9.9 ± 0.2 ^b^
Glucose	2.2 ± 0.7 ^a^	4.2 ± 2.2 ^a^	1.5 ± 0.1 ^a^	2.03 ± 0.04 ^a^
Xylose + Mannose	15.9 ± 0.6 ^a^	7.1 ± 1.8 ^b^	2.6 ± 0.04 ^c^	3.03 ± 0.2 ^c^
Uronic acids	56.4 ± 1.0 ^a^	64.6 ± 4.9 ^a^	66.7 ± 1.4 ^a^	64.5 ± 1.02 ^a^
Pectic neutral sugars *	55.6 ± 4.5 ^b^	63.6 ± 5.9 ^ab^	74.85 ± 0.62 ^a^	76.1 ± 1.3 ^a^
Branching	6	8.9	5.8	6.3

Values represented as averages ± standard deviation. Pectic neutral sugars calculated as (rhamnose + arabinose + galactose) × 100/total neutral sugars. Branching calculated as (rhamnose + arabinose/galactose). * Units expressed as % gram per 100 g sample. ^a^ Within the same row, means with different letters are significantly different at *p* < 0.05.

**Table 3:** Concentration (10^−3^ mol/L) of short chain fatty acids released in the 0.5 M alkaline treated cell-wall polysaccharide samples (NAC, CCN) as compared to glucose, inulin, and rhamnogalacturonan standards which were obtained from *Lactobacillus rhamnosus* GG (R0343 AR) fermentation using different carbon sources. Values determined as difference with concentrations detected immediately after inoculation.

	Time (h)	Glucose	Inulin	RhamnoG	NAC	CCN
Lactic acid	24	313.0 ± 4.0 ^a^	212.5 ± 15.5 ^a^	508.8 ± 10.3 ^a^	427.6 ± 62.0 ^a^	343.0 ± 85.9 ^a^
	48	386.0 ± 16.2 ^a^	315.7 ± 14.8 ^a^	293.5 ± 26.0 ^a^	354.7 ± 41.1 ^a^	393.0 ± 44.2 ^a^
Acetic acid	24	58.1 ± 8.4 ^a^	46.4 *^a^	112.2 ± nd*^a^	42.5 ± 0.9 ^a^	71.9 *^a^
	48	111.7 *^a^	66.4 *^a^	39.0 ± 8.5 ^a^	35.3 ± 3.0 ^a^	96.8 *^a^
Propionic acid	24	4.7 *^a^	1.0 ± 0.04 ^a^	1.8 ± 0.03 ^a^	1.1 ± 0.1 ^a^	1.2 *^a^
	48	0.7 *^b^	0.4 *^b^	0.6 ± 0.06 ^b^	1.1 ± 0.2 ^ab^	1.9 ± 0.3 ^a^
Butyric acid	24	1.8 *^a^	0.3 *^a^	0.4 ± 0.04 ^a^	-	-
	48	-	-	-	-	-
Total	24	377.6	260.2	623.2	471.2	416.1
	48	498.4	382.5	333.1	391.1	491.7

Values represented as average ± standard deviation. RhamnoG; Rhamnogalacturonan. * Standard deviation is zero or not detected. ^a^ Within the same row, means with different letters are significantly different at *p* < 0.05.

**Table 4:** Concentration (10^−3^ mol/L) of short chain fatty acids released in the 0.5 M alkaline treated cell-wall polysaccharide samples (NAC, CCN) as compared to glucose, inulin, and rhamnogalacturonan standards which were obtained from *Bifidobacterium longum* (ATCC 15707^®^) fermentation using different carbon sources. Values determined as difference with concentrations detected immediately after inoculation.

	Time (h)	Glucose	Inulin	RhamnoG	NAC	CCN
Lactic acid	24	31.3 ± 5.1 ^a^	56.3 *^a^	178.0 * ^a^	169.0 ± 2.4 ^a^	273.2 ± 66.4 ^a^
	48	404.4 ± 96.6 ^a^	281.3 ± 1.8 ^a^	327.9 ± 2.1 ^a^	299.8 ± 18.3 ^a^	216.2 ± 1.5 ^a^
Acetic acid	24	44.4 ± 3.9 ^a^	43.7 ± 10.3 ^a^	52.6 *^a^	39.5 *^a^	53.9 *^a^
	48	230.1 ± 11.3 ^a^	55.3 ± 3.6 ^b^	50.5 ± 1.5 ^b^	28.6 ± 2.9 ^b^	53.3 *^b^
Propionic acid	24	1.9 ± 0.4 ^a^	1.3 ± 0.2 ^a^	2.6 ± 0.4 ^a^	1.9 ± 0.2 ^a^	1.7 *^a^
	48	0.6 *^a^	0.5 ± 0.1 ^a^	0.5 ± 0.03 ^a^	0.3 *^a^	2.0 *^a^
Butyric acid	24	0.3 *	0.3 *	-	-	-
	48	-	-	-	-	-
Total	24	77.9	101.6	233.3	210.4	328.8
	48	635.1	337.1	378.9	328.7	271.5

Values represented as average ± standard deviation. RhamnoG; Rhamnogalacturonan. * Standard deviation is zero or not detected. ^a^ Within the same row, means with different letters are significantly different at *p* < 0.05.

## Data Availability

The data presented in this study are available upon request from the corresponding author.
